# Supplementary material for: A Lifetime of a Dispenser-Release Rates of Olive Fruit Fly-Associated Yeast Volatile Compounds and Their Influence on Olive Fruit Fly (Bactrocera oleae Rossi) Attraction
Source: Molecules. 2023 Mar 7;28(6):2431. doi: 10.3390/molecules28062431 (PMC10052186; doi:10.3390/molecules28062431)
Supplement: Supplementary file 1 [file molecules-28-02431-s001.zip › molecules-2256767-supplementary.pdf]

Supplementary Materials

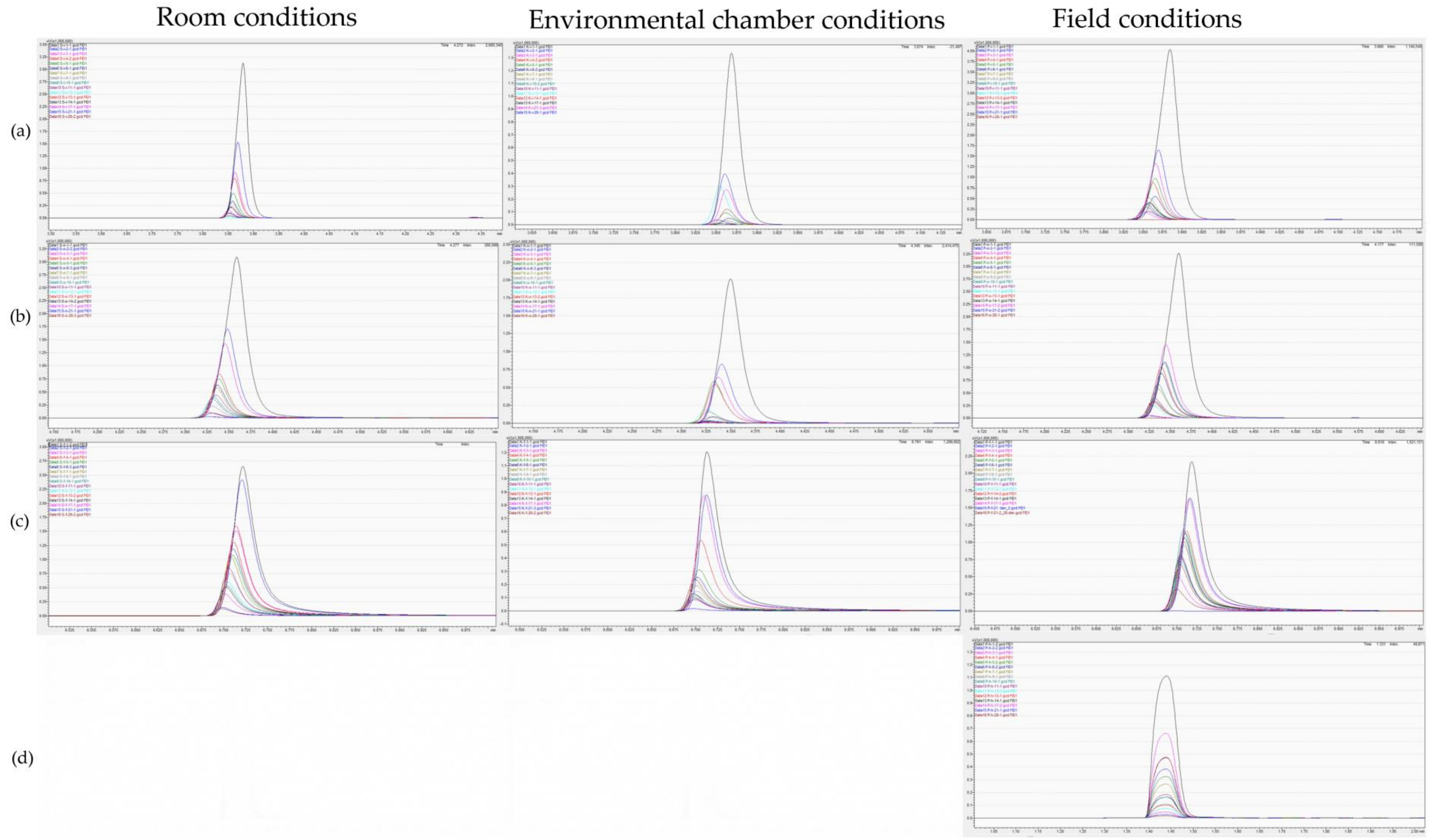

Figure S1: Monitoring of 3 tested OFF-associated yeast volatile compounds during 4 week experiment, isoamyl alcohol (a), 2-octanone (b) and 2-phenethyl acetate (c), under controlled conditions and in the field, and a non-yeast associated volatile compound n-hexane (d) in the field, presented as enlarged chromatograms obtained by analysis using HS-GC/FID.

Table S1: Daily monitoring of individual volatile compound (VOC) weight changes in rubber septa during a 4-week experiment.

	Rubber septa- RS											
	**Room conditions**				**Environmental chamber**				**Field conditions**			
	Isoamyl alcohol	2-Octanone	2-Phenethyl acetate	n-Hexane	Isoamyl alcohol	2-Octanone	2-Phenethyl acetate	n-Hexane	Isoamyl alcohol	2-Octanone	2-Phenethyl acetate	n-Hexane
Before loading of VOC	0.0000	0.0000	0.0000	0.0000	0.0000	0.0000	0.0000	0.0000	0.0000	0.0000	0.0000	0.0000
After loading of VOC	0.0405	0.0410	0.0516	0.0328	0.0405	0.0410	0.0516	0.0328	0.0405	0.0410	0.0516	0.0327
Initial day of measurement	0.0128	0.0134	0.0471	0.0025	0.0149	0.0135	0.0474	0.0018	0.0167	0.0119	0.0483	0.0024
1st day	0.0019	0.0053	0.0388	0.0005	0.0031	0.0072	0.0260	0.0028	0.0023	0.0059	0.0346	0.0011
2nd day	0.0004	0.0029	0.0314	0.0003	0.0030	0.0056	0.0186	0.0031	0.0005	0.0027	0.0241	0.0000
3rd day	0.0001	0.0021	0.0275	-0.0002	0.0030	0.0049	0.0156	0.0031	0.0007	0.0023	0.0205	0.0002
4th day	-0.0001	0.0012	0.0238	-0.0002	0.0032	0.0047	0.0132	0.0035	0.0005	0.0018	0.0154	0.0003
5th day	-0.0001	0.0010	0.0203	-0.0003	0.0036	0.0047	0.0114	0.0039	0.0007	0.0015	0.0114	0.0003
6th day	0.0000	-0.0058	0.0175	-0.0002	0.0037	0.0046	0.0101	0.0040	0.0008	0.0016	0.0104	0.0007
7th day	-0.0002	0.0005	0.0150	-0.0003	0.0034	0.0042	0.0085	0.0037	0.0004	0.0010	0.0082	0.0002
9th day	0.0000	0.0001	0.0117	-0.0004	0.0027	0.0034	0.0061	0.0031	0.0004	0.0044	0.0071	0.0002
10th day	-0.0004	0.0000	0.0109	-0.0005	0.0034	0.0037	0.0066	0.0036	0.0005	0.0009	0.0066	0.0003
11th day	-0.0005	-0.0001	0.0097	-0.0006	0.0032	0.0036	0.0059	0.0035	0.0002	0.0005	0.0056	0.0000
12th day	-0.0004	-0.0002	0.0089	-0.0006	0.0035	0.0048	0.0058	0.0037	0.0001	0.0004	0.0055	0.0002
13th day	-0.0004	-0.0002	0.0079	-0.0006	0.0034	0.0036	0.0052	0.0034	0.0001	0.0004	0.0051	0.0000
14th day	-0.0005	-0.0002	0.0072	-0.0005	0.0028	0.0031	0.0045	0.0028	0.0001	0.0003	0.0046	0.0004
17th day	-0.0006	-0.0205	0.0057	-0.0005	0.0032	0.0034	0.0039	0.0032	0.0000	0.0004	0.0042	0.0001
18th day	-0.0003	-0.0003	0.0052	-0.0005	0.0036	0.0039	0.0045	0.0038	0.0000	0.0000	0.0035	0.0001
21st day	0.0032	0.0031	0.0039	0.0033	0.0032	0.0031	0.0039	0.0033	0.0021	0.0009	0.0036	0.0007
28th day	0.0022	0.0025	0.0020	0.0019	0.0022	0.0025	0.0020	0.0019	0.0004	0.0009	0.0028	0.0007

The results are presented as mean values obtained from three measurements.

Table S2: Daily monitoring of individual volatile compound weight changes in polypropylene vials during the 4-week experiment.

	Polypropylene vials- PPV											
	Room conditions				Environmental chamber				Field conditions			
	Isoamyl alcohol	2-Octanone	2-Phenethyl acetate	n-Hexane	Isoamyl alcohol	2-Octanone	2-Phenethyl acetate	n-Hexane	Isoamyl alcohol	2-Octanone	2-Phenethyl acetate	n-Hexane
Before loading of VOC	0.0000	0.0000	0.0000	0.0000	0.0000	0.0000	0.0000	0.0000	0.0000	0.0000	0.0000	0.0000
After loading of VOC	0.8100	0.8200	1.0320	0.6550	0.8100	0.8200	1.0320	0.6550	0.8100	0.8200	1.0320	0.6550
Initial day of the measurement	0.7938	0.8097	1.0063	0.6316	0.7869	0.8150	1.0261	0.6649	0.7960	0.8096	0.9984	0.6323
1st day	0.7923	0.8083	1.0063	0.3319	0.7924	0.8185	1.0279	0.2366	0.7964	0.8087	0.9995	0.3340
2nd day	0.7890	0.8068	1.0064	0.0626	0.7951	0.8199	1.0286	0.0516	0.7960	0.8076	0.9999	0.0748
3rd day	0.7869	0.8058	1.0066	0.0428	0.7726	0.8201	1.0304	0.0433	0.7955	0.8079	1.0007	0.0534
4th day	0.7845	0.8047	1.0071	0.0355	0.7956	0.8196	1.0331	0.0526	0.7949	0.8072	1.0014	0.0419
5th day	0.7821	0.8040	1.0076	0.0316	0.7949	0.8178	1.0352	0.0378	0.7937	0.8067	1.0023	0.0374
6th day	0.7795	0.8030	1.0079	0.0291	0.8372	0.8136	1.0371	0.0350	0.7947	0.8075	1.0098	0.0376
7th day	0.7767	0.8019	0.0083	0.0265	0.7913	0.8070	1.0383	0.0357	0.7929	0.8061	1.0036	0.0370
9th day	0.7666	0.7977	1.0070	0.0224	0.7858	0.7910	1.0403	0.0377	0.7903	0.8038	1.0036	0.0324
10th day	0.7654	0.7967	1.0069	0.0217	0.7843	0.7857	1.0412	0.0355	0.7902	0.8037	1.0036	0.0327
11th day	0.7619	0.7950	1.0065	0.0207	0.7813	0.7765	1.0420	0.0340	0.7874	0.8014	1.0025	0.0294
12th day	0.7582	0.7934	1.0062	0.0197	0.7785	0.7678	1.0428	0.0332	0.7861	0.8002	1.0022	0.0285
13th day	0.7543	0.7915	1.0055	0.0185	0.7753	0.7578	1.0434	0.0330	0.7835	0.7982	1.0013	0.0262
14th day	0.7509	0.7900	1.0052	0.0178	0.7725	0.7480	1.0439	0.0323	0.7814	0.7965	1.0012	0.0249
17th day	0.7420	0.7858	1.0048	0.0167	0.7617	0.7194	1.0452	0.0311	0.7779	0.7947	1.0008	0.0240
18th day	0.7395	0.7846	1.0048	0.0166	0.7589	0.7104	1.0453	0.0305	0.4431	0.7937	1.0009	0.0238
21st day	0.7338	0.7811	1.0051	0.0165	0.7504	0.6873	1.0452	0.0303	0.7745	0.7926	1.0027	0.0249
28th day	0.7110	0.7247	1.0043	0.0037	0.7180	0.6024	1.0420	0.0262	0.7634	0.7840	1.0002	0.0203

The results are presented as mean values obtained from three measurements.

Table S3: Monitoring of individual volatile compound mass concentration changes in rubber septa during the 4-week experiment.

	**Room conditions**			**Environmental chamber conditions**			**Field conditions**			
Measurement day	Isoamyl alcohol	2-Octanone	2-Phenethyl acetate	Isoamyl alcohol	2-Octanone	2-Phenethyl acetate	Isoamyl alcohol	2-Octanone	2-Phenethyl acetate	n-Hexane
Initial day of the measurement*	2.7408	3.881	7.2227	2.7408	3.881	7.2227	2.7408	3.881	7.2227	0.7673
1st day	0.3158	0.3321	6.9932	0.1341	0.2106	3.1074	0.3898	0.3617	6.4811	0.2278
2nd day	0.1227	0.1736	6.5548	0.0276	0.1174	2.1123	0.1148	0.1634	4.3648	0.1120
3rd day	0.0822	0.1390	4.7725	0.0154	0.0763	1.7076	0.1024	0.1491	3.7576	0.0993
4th day	0.0624	0.1079	3.6901	0.0091	0.0617	1.3122	0.0593	0.1129	2.7455	0.0745
5th day	0.0364	0.0680	2.9952	0.0069	0.0506	0.8147	0.0596	0.0908	2.5770	0.0711
6th day	0.0261	0.0562	2.8771	0.0050	0.0156	0.6409	0.0467	0.0946	2.2021	0.0644
7th day	0.0227	0.0495	2.2945	0.0027	0.0156	0.5020	0.0356	0.0427	2.2155	0.0659
9th day	0.0220	0.0318	1.9131	0.0007	0.0110	0.3277	0.0406	0.0336	2.0094	0.0327
10th day	0.0169	0.0303	1.7788	0.0009	0.0108	0.2994	0.0297	0.0324	1.9706	0.0308
11th day	0.0165	0.0196	1.7717	0.0007	0.0024	0.2442	0.0255	0.0300	1.6663	0.0331
12th day	0.0005	0.0209	1.7245	0.0201	0.0023	0.2425	0.0208	0.0229	1.6751	0.0138
13th day	0.0133	0.0221	1.7899	0.0006	0.0050	0.2498	0.0207	0.0200	1.6575	0.0304
14th day	0.0114	0.0149	1.6261	0.0003	0.0032	0.2263	0.0236	0.0253	1.6462	0.0248
17th day	0.0114	0.0085	0.9462	0.0001	0.0024	0.1156	0.0155	0.0221	1.1712	0.0095
21st day	0.0066	0.0035	0.6486	0.0001	0.0006	0.2917	0.0168	0.0067	0.7132	0.0070
28th day	0.0080	0.0038	0.2987	0.0036	0.0010	0.0617	traces	0.0006	0.0134	0.0012

*measured concentration 24h after loading and absorbing of volatile compound in rubber septa dispenser

The results are presented as mean values obtained from three measurements.

Table S4: Validation parameters for tested volatile compounds

**Compound**	**Retention time (min)**	**Concentration range (ppm)**	**Slope**	**Intercept**	**Coefficient of determination (R2)**
n-Hexane	1.37	0.033-6.55	17.366	-0.0245	0.9985
Isoamyl alcohol	3.75	0.051-8.1	17.06	0.7177	0.9987
2-Octanone	4.23	0.052-8.19	15.708	-0.393	0.9992
2-Phenethyl acetate	6.57	0.103-10.32	0.7848	0.013	0.9972
